# Association of Occupational Pesticide Exposure With Immunochemotherapy Response and Survival Among Patients With Diffuse Large B-Cell Lymphoma

**DOI:** 10.1001/jamanetworkopen.2019.2093

**Published:** 2019-04-19

**Authors:** Sylvain Lamure, Camille Carles, Quam Aquereburu, Philippe Quittet, Emmanuelle Tchernonog, Franciane Paul, Eric Jourdan, Agathe Waultier, Christine Defez, Ihssen Belhadj, Laurence Sanhes, Sara Burcheri, Daniel Donadio, Carole Exbrayat, Alain Saad, Jean-Luc Labourey, Isabelle Baldi, Guillaume Cartron, Pascale Fabbro-Peray

**Affiliations:** 1 Department of Clinical Hematology, Montpellier University Hospital, Montpellier, France; 2 Hematology Federation of University Hospitals of Montpellier and Nîmes, University of Montpellier, Montpellier, France; 3 Institute of Public Health, Epidemiology, and Development, University of Bordeaux, Bordeaux, France; 4 Department of Clinical Hematology, Gard Institute of Cancer, Nîmes University Hospital, University of Montpellier, Nîmes, France; 5 Department of Biostatistics, Clinical Epidemiology, Public Health, and Innovation in Methodology, Nîmes University Hospital, University of Montpellier, Montpellier, France; 6 Clinique du Parc, Castelneau-Le-Lez, Montpellier, France; 7 Department of Clinical Hematology, Perpignan General Hospital, Perpignan, France; 8 Department of Clinical Hematology, Béziers General Hospital, Béziers, France; 9 Department of Clinical Oncology, Carcassonne General Hospital, Carcassonne, France; 10 Laboratory of Biostatistics Epidemiology and Clinical Research, University of Montpellier, Montpellier, France

## Abstract

**Importance:**

Professional use of pesticides is a risk factor for non-Hodgkin lymphoma. The main biological mechanisms of pesticides and chemotherapy are genotoxicity and reactive oxygen species generation. Cellular adaptation among patients exposed to low doses of genotoxic and oxidative compounds might hinder chemotherapy efficiency in patients with lymphoma.

**Objective:**

To examine the association of occupational exposure to pesticides with immunochemotherapy response and survival among patients treated for diffuse large B-cell lymphoma.

**Design, Setting, and Participants:**

This retrospective cohort study assessed patients treated from July 1, 2010, to May 31, 2015, for diffuse large B-cell lymphoma, with a 2-year follow-up. The study took place at 6 university and nonuniversity hospitals in Languedoc-Roussillon, France. A total of 404 patients with newly diagnosed diffuse large B-cell lymphoma treated with anthracycline-based immunochemotherapy were included before the study began. Occupational history was reconstructed for 244 patients and analyzed with the PESTIPOP French job-exposure matrix to determine likelihood of occupational exposure to pesticides. Analysis of the data was performed from July 15, 2017, to July 15, 2018.

**Main Outcomes and Measures:**

Treatment failure (ie, partial response, stable disease, disease progression, or interruption for toxic effects) rate, 2-year event-free survival, and overall survival between exposed and nonexposed patients after adjustment for confounding factors.

**Results:**

A total of 244 patients (mean [SD] age, 61.3 [15.2] years; 153 [62.7%] male) had complete occupational data. Of these patients, 67 (27.4%) had occupational exposure to pesticides, with 38 exposed through agricultural occupations. Occupational exposure was not associated with clinical and biological characteristics at diagnosis. Occupationally exposed patients had a significantly higher treatment failure rate (22.4% vs 11.3%; *P* = .03; adjusted odds ratio [AOR] for confounding factors, 3.0; 95% CI, 1.3-6.9); this difference was higher among patients with exposing agricultural occupations compared with other patients (29.0% vs 11.7%; AOR, 5.1; 95% CI, 2.0-12.8). Two-year event-free survival was 70% in the occupationally exposed group vs 82% in the unexposed group (adjusted hazard ratio [AHR] for confounding factors, 2.2; 95% CI, 1.3-3.9). Among patients with exposing agricultural occupations compared with other patients, the difference was more pronounced (2-year event-free survival, 56% vs 83%; AHR, 3.5; 95% CI, 1.9-6.5). Similarly, 2-year overall survival was lower in the group of patients with exposing agricultural occupations compared with other patients (81% vs 92%; AHR, 3.9; 95% CI, 1.5-10.0).

**Conclusions and Relevance:**

This retrospective study showed that agricultural occupational exposure to pesticides was associated with treatment failure, event-free survival, and overall survival among patients with diffuse large B-cell lymphoma.

## Key Points

**Question:**

What is the prognosis of patients treated for diffuse large B-cell lymphoma (DLBCL) with a history of occupational exposure to pesticides?

**Findings:**

In this cohort study of 244 patients treated for DLBCL, 67 patients were exposed to pesticides, with 38 exposed through agricultural occupations. The treatment failure rate was significantly higher among exposed patients compared with nonexposed patients after adjusting for potential confounding factors; the association was stronger when patients with exposing agricultural occupations were compared with those with nonexposing occupations.

**Meaning:**

Professional exposure to pesticides may be an independent risk factor for treatment failure in DLBCL.

## Teaser

This cohort study examines the association of occupational exposure to pesticides with immunochemotherapy response and survival in patients in France treated for diffuse large B-cell lymphoma (DLBCL).

## Introduction

There is an increasing body of evidence that different pesticides or residues used in agricultural occupations are a risk factor for non-Hodgkin lymphoma (NHL), including diffuse large B-cell lymphoma (DLBCL). Occupational exposure has been reported to be associated with a higher rate of NHL in several meta-analyses^1,2,3,4,5^ and prospective studies.^6,7^ More recently, a prospective study^8^ showed an association of a higher frequency of organic food intake with decreasing risk of NHL. Three agents have been associated with NHL and classified as carcinogenic by the International Agency for Research on Cancer: glyphosate, malathion, and diazinon.^9^ Fungicides, insecticides, or herbicides are widely used for farming; their cumulated toxic effects, called the *cocktail effect*, are not well characterized and may induce specific carcinogenic pathways.^10^

Diffuse large B-cell lymphoma accounts for approximately 30%^11^ of NHL, characterized by an aggressive presentation. In a prospective study,^6^ the relative standardized incidence ratio for DLBCL among farmers using pesticides was 1.3 (95% CI, 1.0-1.6) and 1.5 (95% CI, 1.1-2.1) in spouses. Little is known about pesticide-related DLBCL regarding clinical and pathologic characteristics and prognosis. Treatment of DLBCL is anthracycline-based chemotherapy combined with an anti-CD20 monoclonal antibody, allowing a high rate of complete response (2-year progression-free survival of 69% and 2-year overall survival [OS] of 78%).^12^ The outcome for primary refractory or early relapsing DLBCL is poor: 20% of patients are alive after 2 years according to a recent retrospective study.^13^

Genotoxicity and reactive oxygen species generation in healthy cells and cancer cells are common mechanisms of action shared by different pesticides used for agricultural production and chemotherapy. They are involved in the transformation of healthy lymphocytes into clonal ones after different pesticide exposure.^14,15^ Adaptation of different cell systems after different pesticide exposure could lead to DNA repair and antioxidant mechanisms. These mechanisms represent a significant pathway for chemotherapy resistance: a DNA repair score was correlated significantly with outcome after chemotherapy for DLBCL.^16^ Another study^17^ found an association between response to chemotherapy and antioxidant pathways in lymphoid malignant tumors, and one study^18^ found resistance of lymphoma cells to cytotoxic treatment after paraquat exposure.

We hypothesized that cellular adaptation to damage induced by long-term occupational pesticide exposure, promoting DNA repair pathways and antioxidant defenses, hinders chemotherapy efficiency. The aim of this study was to examine the association between occupational exposure to pesticides among patients treated for DLBCL and immunochemotherapy response and survival.

## Methods

### Study Design

A retrospective, multicenter cohort of adults with DLBCL receiving first-line immunochemotherapy was performed. Comparison of patients exposed to pesticides for professional activity with nonexposed patients was performed. This report followed the Strengthening the Reporting of Observational Studies in Epidemiology (STROBE) reporting guideline. Ethical and regulatory approvals were obtained from the French Data Protection Committees (Advisory Committee on Information Processing in Material Research in the Field of Health and National Commission for Computing and Liberties). No informed consent was required but oral information was given to the patients or their proxies. Data were deidentified when collected.

### Setting

All patients treated for DLBCL from January 1, 2010, and July 15, 2015, in the 6 hematology departments of Languedoc-Roussillon, France (2 university hospitals, 1 private hospital, and 3 general hospitals) were considered for inclusion. Analysis of the data was performed from July 1, 2017, to July 15, 2018.

### Participants

Patients older than 18 years with DLBCL receiving standardized first-line treatment were eligible for inclusion. We performed a systematic review of the national prospective payment system files of patients with non-Hodgkin nonfollicular lymphoma. Inclusion criteria were histopathologic diagnosis of DLBCL and anthracycline-based and anti-CD20–based first-line therapy. Exclusion criteria were tutorship, risk factors linked to immune dysfunction (ie, HIV infection, hepatitis C virus infection, rheumatoid arthritis, systemic lupus erythematous, Sjögren syndrome, psoriasis, celiac disease, and common variable immunodeficiency), primary central nervous system lymphoma, leg-type B-cell lymphoma, lymphomatoid granulomatosis, DLBCL related to inflammation or transplantation, plasmablastic lymphoma, primary effusion lymphoma, Castleman disease–associated DLBCL, double-hit lymphoma, and unclassified lymphoma. Contact with patients or their proxies was made by telephone, and definitive inclusion was made after patient or proxy agreement and survey completion. Patients from Montpellier University Hospital were the first recruited, and the yearly rate of unreachable patients was used to adjust the period of preselection for the other centers to include the necessary number of participants required for the study. Clinical data for outcomes were collected at least 2 years after diagnosis. Vital status of patients unavailable to follow-up was investigated on the civil register.

### Diagnostic Criteria

Histopathologic criteria for DLBCL are defined in the World Health Organization 2008 classification of lymphoid neoplasm.^11^ Complete clinical, biological, and imagery-related diagnostic criteria, as well as response and follow-up, were retrieved from patient files.

### Outcomes

The primary outcome was response to treatment assessed by local investigators after 4 cycles of immunochemotherapy, clinical evaluation, and positron emission tomography or computed tomography if not available. Treatment failure was defined as absence of complete response according to the 2014 Lugano criteria.^19^ Treatment discontinued because of toxic effects was classified as treatment failure. Secondary outcomes were event-free survival (EFS), defined as the absence of progressive disease during treatment, disease activity after 4 cycles of treatment, relapse, or toxicity-related death, and OS was defined as the absence of death within the 2 years after diagnosis.

### Pesticide Exposures

Data on job positions held longer than 1 year were collected. The corresponding codes according to French classification for socioeconomic position^20^ and economic activities^21^ were inputted into the French job exposure matrix (JEM) PESTIPOP^22^ to assess the likelihood of pesticide exposure and its reliability (eAppendix in the Supplement). Source of exposure was classified into 4 occupational groups: agriculture (eg, vineyard workers), green spaces (eg, gardeners), woodwork (eg, carpenters), and public hygiene (eg, pest control workers) (patients could be placed into >1 group). Occupational pesticide exposure was considered in 4 different ways. The first categorization considered all probabilities of exposure. The second one considered only exposing agricultural occupations. The third one considered only high probability of exposure (>75%), as defined by the PESTIPOP matrix generation. The fourth considered high probability of exposure (>75%) and high reliability as defined by the PESTIPOP matrix generation.

### Potential Confounders and Other Prognostic Factors

Prognostic factors were recorded from patient files for multivariate analysis: age, European Cooperative Oncology Group score, lactate dehydrogenase level, Ann Arbor stage, extranodal disease (combined with the International Prognostic Index [IPI]),^23^ bulky disease (tumor size >10 cm), prior tumor treated with chemotherapy or radiotherapy, transformation from low-grade lymphoma, treatment in a teaching hospital, marital status, and travel time to hospital less than 15 minutes, according to categories defined by Le Guyader-Peyrou.^24^ The distance between health care centers and home was computed on the basis of Open Street Resource Map geographic data^25^ and using an R package interface rCarto (R Foundation).^26^

### Data Source and Measurement

A single investigator (S.L.) collected clinical data and outcomes from patient files in hospitals at least 2 years after diagnosis. A clinical research assistant (Q.A.) performed data collection of occupational history in 3 steps: (1) informing patients or relatives of the study purpose by telephone, (2) mailing a survey for occupational history, and (3) data collection during the second telephone interview. For each job, data collected were job title, description of tasks, and periods of work. Job titles were encoded according to the French classifications.^20,21^ The anonymized encoded job data were sent to an independent epidemiologic team, who performed computational analysis using JEM masked for all patient information.

### Bias

Selection bias was limited by prescreening all patients treated for lymphoma in the 6 hospitals. Differential classification bias was limited through 2 masking levels. Investigation for occupational history was performed masked for primary outcome but not for OS because of telephone contact with patients or proxies. Classification for exposure to pesticides was performed masked for all outcomes by an independent team. Bias attributable to confounding was controlled using a multivariate model for statistical analysis, with all independent prognostic factors described in the literature^23,24^ and potential confounding factors highlighted by univariate analysis.

### Study Size

According to a prospective cohort of patients with hematologic disorder conducted at Montpellier University Hospital, approximately 15% of patients with DLBCL were farmers, and the treatment failure rate of nonexposed patients was approximately 25%. We hypothesized a doubled treatment failure rate in pesticide-exposed patients. With use of a relative risk with a 2-sided α risk of 5% and a power of 80%, the number of patients required to meet significance was 238, of whom 34 would have been exposed to pesticides.

### Statistical Analysis

#### Univariate Analysis of Confounding Factors

Confounding factors were compared according to pesticide exposure treatment failure by the χ^2^ test or Fisher exact test, as appropriate, for qualitative factors; the *t* test or Mann-Whitney test according to the distribution of the variables for quantitative factors, according to censored data (survival) by log-rank test for qualitative variables; and univariate Cox proportional hazards regression for discrete or continuous variables.

#### Outcome Analysis

The treatment failure rate was compared between exposed and nonexposed patients using unconditional logistic regression, providing unadjusted odds ratios (ORs) and adjusted ORs (AORs) with 95% CIs (Wald test). Adjustment for known prognostic factors was systematically performed. Factors associated with treatment failure with a univariate *P* < .20 were considered as potential confounding factors for multivariate analysis. For model building, we applied a change-in-estimate criterion, which involves looking separately at the OR compared with the AOR for each single variable. If the unadjusted and adjusted values differed by more than 10% (ie, AOR or OR >1.1 or <0.9), the variable was included in the multivariable model. The Hosmer-Lemeshow test was used to check the fit of the model.

The OS and EFS were described by Kaplan-Meier curves and compared between exposed and nonexposed patients by log-rank test and Cox proportional hazards regression model, providing unadjusted hazard ratios (HRs) and adjusted hazard ratios (AHRs) with 95% CIs (Wald test). Model building was made as described above. The OS was calculated from diagnosis to death (event data) or last date of follow-up (censored data). The EFS was calculated from diagnosis to first evidence of active disease under treatment or relapse (event data) or last date of follow-up without active disease (censored data).

SAS statistical software, version 9.4 (SAS Institute Inc) was used to perform all statistical analysis.

## Results

### Participants

**Figure 1.**
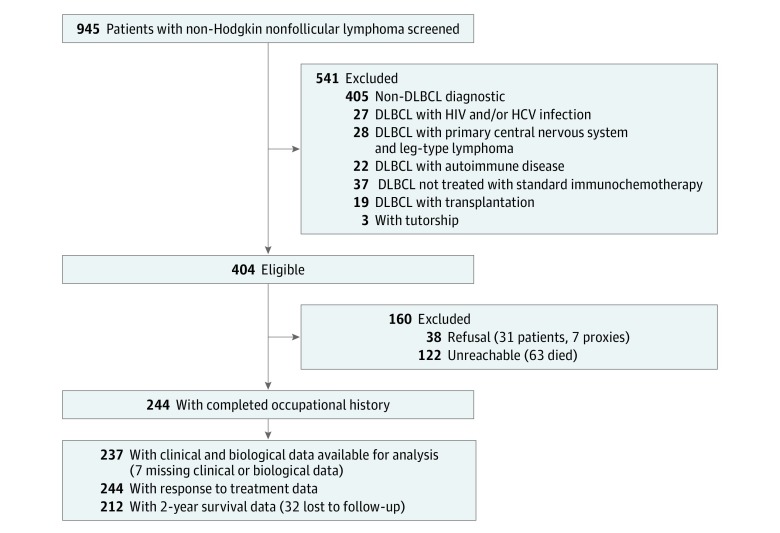
Patient Flowchart Demographic data were missing for 50 patients. DLBCL indicates diffuse large B-cell lymphoma; HCV, hepatitis C virus.

In total, 244 patients (mean [SD] age, 61.3 [15.2] years; 153 [62.7%] male) were included and assessed for analysis, with a median length of follow-up of 33 months. A total of 945 patient files were screened. First inclusions were made in Montpellier, France, from January 1, 2010, to July 15, 2015. The rate of unreached patients before 2012 was greater than 60%; thus, for all other centers, patients diagnosed with non-Hodgkin nonfollicular lymphoma from January 1, 2012, to July 15, 2015, were screened: 404 met the inclusion criteria, of whom 282 were successfully contacted, with 38 refusing inclusion. Among the 122 unreached patients, 63 were confirmed to have died (Figure 1).

Comparison of characteristics between the included and not included (unreached or refusal to answer the job history interview) groups revealed a higher rate of unfavorable IPI in the not included group (44.3% vs 18.6%, *P* < .001). Outcomes were worse in not included patients (treatment failure, 43.1% vs 14.3%, *P* < .001). Two-year EFS was lower for not included patients (53.2% [95% CI, 44.8%-60.9%] vs 78.0% [95% CI, 72.1%-82.8%]) as was 2-year OS (60.1% [95% CI, 51.7%-67.6%] vs 89.8% [95% CI, 85.1%-93.0%]) (eTable 1 in the Supplement). Among interview responders, 198 (81.1%) were patients and 46 (18.9%) were proxies.

### Descriptive Data

The IPI was favorable for 83 patients (35.0%), intermediary I and II for 55 (23.2%) each, and unfavorable for 44 (18.6%); 35 patients (14.3% of 194 known status) were living alone, 151 (61.9%) were treated in teaching hospitals, and travel time to the hospital was less than 15 minutes for 37 patients (15.2%). The main treatment was rituximab, cyclophosphamide, doxorubicin, vincristine, and prednisone (R-CHOP) for 214 patients (87.7%), mini-R-CHOP (reduced R-CHOP dosage) for 25 patients (10.2%), and doxorubicin, cyclophosphamide, vindesine, bleomycin, prednisone, and rituximab (R-ACVBP) for 5 (2%).

No difference in demographic characteristics, initial presentation, IPI, and treatment protocol between exposed and nonexposed patients was found; patients with no agricultural exposure were more likely to have disseminated disease (Ann Arbor stage IV) (104 [50.5%]) compared with patients with agricultural exposure (13 [34.2%]) (*P* = .05) (Table 1).

**Table 1.** Patient Characteristics by Exposure to Pesticides^a^

Characteristic	Occupational Exposure (n = 67)	No Occupational Exposure (n = 177)	*P* Value	Exposing Agricultural Occupations (n = 38)	No Exposing Agricultural Occupations vs Others (n = 206)	*P* Value	High Probability of Occupational Exposure (n = 48)	No High Probability of Occupational Exposure (n = 196)	*P* Value	High Probability and Reliability of Occupational Exposure (n = 35)	No High Probability and Reliability of Occupational Exposure (n = 209)	*P* Value
Age, mean (SD), y	60.8 (15.8)	61.4 (15.4)	.77	64.3 (15.3)	60.7 (15.5)	.19	61.1 (16.7)	61.3 (15.2)	.92	63.5 (15.9)	60.9 (15.4)	.36
Male	44 (65.7)	109 (61.6)	.56	26 (68.4)	127 (61.6)	.43	33 (68.8)	120 (61.2)	.33	22 (62.9)	131 (62.7)	.98
Previous cancer												
No	61 (91.0)	152 (85.9)	.55	34 (89.5)	179 (86.9)	.83	44 (91.7)	169 (86.2)	.71	31 (88.6)	182 (87.1)	>.99
Treated with radiotherapy or chemotherapy	2 (3.0)	9 (5.1)		1 (2.6)	10 (4.6)		3 (6.2)	17 (8.7)		3 (8.6)	17 (8.1)	
Not treated with radiotherapy or chemotherapy	4 (6.0)	16 (9.4)		3 (7.9)	17 (8.3)		1 (2.1)	10 (5.1)		1 (2.8)	10 (4.8)	
ECOG score												
No. of patients	64	176		37	203		47	193		35	205	
0	21 (32.8)	57 (32.4)	.75	11 (29.7)	67 (33.0)	.60	14 (29.8)	64 (33.2)	.76	11 (31.4)	67 (32.7)	.79
1	25 (39.0)	74 (42.0)		14 (37.8)	85 (41.9)		19 (40.4)	80 (41.4)		14 (40.0)	85 (41.5)	
2	11 (17.2)	21 (11.9)		8 (21.6)	24 (11.8)		9 (19.1)	23 (11.9)		7 (20.0)	25 (12.2)	
3	5 (7.8)	13 (7.4)		2 (5.4)	16 (7.9)		3 (6.4)	15 (7.8)		2 (5.7)	16 (7.8)	
4	2 (3.1)	11 (6.2)		2 (5.4)	11 (5.4)		2 (4.2)	11 (5.7)		1 (2.9)	12 (5.8)	
Ann Arbor staging,												
I	22 (32.9)	34 (19.2)	.05	13 (34.2)	43 (20.9)	.05	16 (33.3)	40 (20.4)	.14	10 (28.6)	46 (22.0)	.34
II	10 (14.9)	30 (17.0)		4 (10.5)	36 (17.5)		6 (12.5)	34 (17.4)		5 (14.3)	35 (16.7)	
III	11 (16.4)	20 (11.3)		8 (21.1)	23 (11.2)		8 (16.7)	23 (11.7)		7 (20.0)	24 (11.5)	
IV	24 (35.8)	93 (52.5)		13 (34.2)	104 (50.5)		18 (37.5)	99 (50.5)		13 (37.1)	104 (49.8)	
Determination												
Bone marrow	3 (4.5)	19 (10.7)	.13	1 (2.6)	21 (10.2)	.22	2 (4.2)	20 (10.2)	.27	1 (2.9)	21 (10.0)	.22
Central nervous system	0	3 (1.7)	.56	0	3 (1.5)	1	0	3 (1.5)	1	0	3 (1.4)	1
Lung	6 (9.0)	18 (10.2)	.78	2 (5.3)	22 (10.7)	.39	3 (6.3)	21 (10.7)	.43	1 (2.9)	23 (11.0)	.22
Liver	4 (6.0)	19 (10.7)	.26	4 (10.5)	19 (9.2)	.77	4 (8.3)	19 (9.7)	1	2 (5.7)	21 (10.0)	.55
Gut	3 (4.5)	17 (9.6)	.19	2 (5.3)	18 (8.7)	.75	2 (42.2)	18 (9.2)	.38	1 (2.9)	19 (9.1)	.32
B symptoms^b^	16 (23.9)	43 (24.3)	.95	7 (18.4)	52 (25.2)	.37	10 (20.8)	49 (25.0)	.54	7 (20.0)	52 (24.9)	.53
Bulky disease	14 (20.9)	45 (25.4)	.46	7 (18.4)	52 (25.2)	.37	11 (22.9)	48 (24.5)	.82	6 (17.1)	53 (25.4)	.29
LDH												
No. of patients	63	171		36	198		45	191		32	202	
LDH greater than normal	37 (58.7)	98 (57.3)	.85	21 (58.3)	114 (57.6)	.92	27 (60.0)	108 (56.5)	.73	19 (59.4)	116 (57.4)	.84
IPI												
No. of patients	63	174		36	201		45	192		32	205	
Favorable (0-1)	24 (38.1)	59 (33.9)	.91	11 (30.6)	72 (35.8)	.95	16 (35.5)	67 (34.9)	.68	10 (31.2)	73 (35.6)	.97
Intermediary I (2)	14 (22.2)	41 (23.6)		9 (25.0)	46 (22.9)		8 (17.7)	47 (24.5)		8 (25.0)	47 (22.9)	
Intermediary II (3)	13 (20.6)	42 (24.1)		9 (25.0)	46 (22.9)		13 (28.9)	42 (21.9)		8 (25.0)	47 (22.9)	
Unfavorable (4-5)	12 (19.1)	32 (18.3)		7 (19.4)	37 (18.4)		8 (17.8)	36 (18.7)		6 (18.8)	38 (18.5)	
Transformation									.			
No. of patients	63	171		36	198		46	188		34	200	
From low-grade lymphoma	8 (12.7)	23 (13.5)	.88	3 (8.3)	28 (14.1)	.34	4 (8.7)	27 (14.4)	.31	4 (11.8)	27 (13.5)	>.99
Main treatment												
R-CHOP	57 (85.1)	157 (88.7)	.60	31 (81.6)	183 (88.8)	.13	40 (83.3)	174 (88.8)	.15	28 (80.0)	185 (88.5)	.13
Mini-R-CHOP	9 (13.4)	16 (9.0)		7 (18.4)	18 (8.7)		8 (16.7)	17 (8.7)		7 (20.0)	19 (9.1)	
R-ACVBP	1 (1.5)	4 (2.3)		0	5 (2.4)		0	5 (2.5)		0	5 (2.4)	
Teaching hospital	40 (59.7)	111 (62.7)	.67	21 (55.3)	130 (63.1)	.36	29 (60.4)	122 (62.2)	.82	20 (57.1)	131 (62.7)	.53
Marital status												
No.	54	140		32	162		40	154		28	166	
Alone	9 (16.7)	26 (18.6)	.76	4 (12.5)	31 (19.1)	.37	6 (15.0)	29 (18.8)	.57	4 (14.3)	31 (18.7)	.58
Travel time to reference hospital, min												
≤15	8 (11.9)	29 (16.4)	.51	2 (5.3)	35 (17.0)	.18	2 (4.2)	35 (17.9)	.02	2 (5.7)	35 (16.8)	.24
16-44	37 (55.2)	101 (57.1)		24 (63.2)	114 (55.3)		27 (56.2)	111 (56.6)		22 (62.9)	116 (55.5)	
≥45	22 (32.8)	47 (26.5)		12 (31.6)	57 (27.7)		19 (39.6)	50 (25.5)		11 (31.4)	58 (27.8)	

Abbreviations: ECOG, European Cooperative Oncology Group; IPI, International Prognostic Index; LDH, lactate dehydrogenase; R-ACVBP, doxorubicin, cyclophosphamide, vindesine, bleomycin, prednisone, and rituximab; R-CHOP, rituximab, cyclophosphamide, doxorubicin, vincristine, and prednisone.

^a^ Data are presented as number (percentage) of patients unless other wise indicated.

^b^ General symptoms, including fever, profuse sweats, and weight loss.

Among the 67 patients (27.4%) exposed to pesticides, 38 had occupational exposure from agriculture, 16 from green spaces maintenance, 15 from wood activities, and 11 from hygiene activities. Fifty-five patients had 1 source of exposure, 11 had 2 sources, and 1 had 3 sources.

Probabilities and duration of pesticide exposure among exposed patients, assessed by PESTIPOP, are given in eTable 2 in the Supplement. Patients with agricultural occupations had nearly 100% probability of exposure. Patients with other exposing occupations had more variable probability of exposure.

### Primary Outcome

Treatment failure occurred in 35 patients (14.3%), including toxicity-related death in 4 patients (1.6%). Two-year EFS was 78% (95% CI, 72%-83%), and 2-year OS was 90% (95% CI, 85%-93%). Mean (SD) age was 61.5 (15.0) years in the treatment failure group vs 61.2 (15.6) years in the treatment response group (*P* = .94). In univariate analysis, lung and liver determination, bulky disease, and IPI were significantly associated with treatment failure (Table 2). Patients professionally exposed to pesticides had higher treatment failure rates compared with nonexposed patients (occupational exposure failure rate, 22.4% vs 11.3%; OR, 2.3; 95% CI, 1.1-4.7; *P* = .03; AOR for confounding factors, 3.0; 95% CI, 1.3-6.9). Patients with exposing agricultural occupations had a higher failure rate compared with others (29.0% vs 11.7%; OR, 3.1; 95% CI, 1.3-7.0; *P* = .005). Considering probability of exposure and reliability of PESTIPOP data, sensitivity analysis was performed. Patients with high probability of occupational exposure had a higher failure rate compared with others (25.0% vs 11.7%; OR, 2.5; 95% CI, 1.1-5.5; *P* = .02). Patients with high probability and reliability of occupational exposure had a higher failure rate compared with others (12.9% vs 22.9%; OR, 2.0; 95% CI, 0.8-4.9; *P* = .13). There was no statistical association between other exposing professions and treatment failure (3 [20.0%] wood exposed vs 32 [14.0%] not wood exposed, 3 [18.8%] green space exposed vs 32 [14.0%] not green space exposed, and 1 [9.0%] hygiene exposed vs 34 [14.6%] not hygiene exposed) (Table 2).

**Table 2.** Univariate and Multivariate Logistic Regression Analysis for Treatment Failure With Exposure to Pesticides^a^

Characteristic	Treatment Failure Rate, No./Total No. (%)	Univariate OR (95% CI)	*P* Value	Multivariate Occupational Exposure (Model 1), AOR (95% CI)^b^	*P* Value	Multivariate Exposing Agricultural Occupations (Model 2), AOR (95% CI)^c^	*P* Value	Multivariate High Probability of Occupational Exposure (Model 3), AOR (95% CI)^d^	*P* Value	Multivariate High Probability and Reliability of Occupational Exposure (Model 4) AOR (95% CI)^e^	*P* Value
Sex											
Male	19/153 (12.4)	1 [Reference]	.27	NA	NA	NA	NA	NA	NA	NA	NA
Female	16/91 (17.6)	1.5 (0.7-3.1)		NA	NA	NA	NA	NA	NA	NA	NA
Previous cancer											
No	29/213 (13.6)	1 [Reference]	.35	NA	NA	NA	NA	NA	NA	NA	NA
Treated with radiotherapy or chemotherapy	5/20 (25.0)	2.1 (0.7-6.3)		NA	NA	NA	NA	NA	NA	NA	NA
Not treated with radiotherapy or chemotherapy	1/11 (9.1)	0.6 (0.1-5.1)		NA	NA	NA	NA	NA	NA	NA	NA
Determination											
No bone marrow	32/222 (14.4)	1 [Reference]	.92	NA	NA	NA	NA	NA	NA	NA	NA
Bone marrow	3/22 (13.6)	1.1 (0.3-3.8)		NA	NA	NA	NA	NA	NA	NA	NA
No CNS	35/241 (14.5)	1 [Reference]	.99	NA	NA	NA	NA	NA	NA	NA	NA
CNS	0/3	NA		NA	NA	NA	NA	NA	NA	NA	NA
No lung	27/220 (12.3)	1 [Reference]	.008	NA	.04	1 [Reference]	.05	1 [Reference]	.05	1 [Reference]	.04
Lung	8/24 (33.3)	3.6 (1.4-9.1)		NA		3.0 (1.0-8.7)		3.0 (1.0-8.6)		3.3 (1.1-9.5)	
No liver	27/221 (12.2)	1 [Reference]	.006	1 [Reference]	.03	NA	NA	NA	NA	1 [Reference]	.05
Liver	8/23 (34.8)	3.8 (1.5-9.9)		3.0 (1.0-8.7)		NA	NA	NA	NA	3.1 (1.0-9.1)	
No gut	34/224 (15.2)	1 [Reference]	.24	NA	NA	NA	NA	NA	NA	NA	NA
Gut	1/20 (5.0)	0.3 (0.0-2.3)		NA	NA	NA	NA	NA	NA	NA	NA
Bulky disease											
No	21/185 (11.3)	1 [Reference]	.02	NA	NA	1 [Reference]	.09	NA	NA	1 [Reference]	.14
Yes	14/59 (23.7)	2.4 (1.2-5.2)		NA	NA	2.1 (0.9-5.0)		NA	NA	1.9 (0.8-4.5)	
IPI											
Favorable and intermediary I (0-2)	9/138 (6.5)	1 [Reference]	.001	1 [Reference]	.02	1 [Reference]	.02	1 [Reference]	.02	1 [Reference]	.02
Intermediary II (3)	11/55 (20.0)	3.6 (1.4-9.2)		3.1 (1.2-8.6)		3.0 (1.1-8.2)		3.1 (1.2-8.3)		2.4 (0.9-6.8)	
Unfavorable (4-5)	12/44 (27.3)	5.4 (2.1-13.9)		4.4 (1.6-12.2)		4.0 (1.4-11.2)		4.3 (1.6-12.0)		3.0 (1.0-8.6)	
Teaching hospital											
Yes	22/151 (14.6)	1 [Reference]	.90	NA	NA	NA	NA	NA	NA	NA	NA
No	13/93 (14.0)	1.0 (0.5-2.0)		NA	NA	NA	NA	NA	NA	NA	NA
Marital status											
Not alone	22/159 (13.8)	1	.36	NA	NA	NA	NA	NA	NA	NA	NA
Alone	7/35 (20.0)	1.6 (0.6-4.0)		NA	NA	NA	NA	NA	NA	NA	NA
Unknown	50	NA		NA	NA	NA	NA	NA	NA	NA	NA
Travel time to hospital, min											
≤15	2/37 (5.4)	1	.26	NA	NA	NA	NA	NA	NA	NA	NA
16-44	21/138 (15.2)	3.1 (0.7-14.1)		NA	NA	NA	NA	NA	NA	NA	NA
≥45	12/69 (17.4)	3.7 (0.8-17.5)		NA	NA	NA	NA	NA	NA	NA	NA
Pesticide exposure											
No exposed occupation	20/177 (11.3)	1 [Reference]	.03	1 [Reference]	.009	NA	NA	NA	NA	NA	NA
All exposed occupation	15/67 (22.4)	2.3 (1.1-4.7)		3.0 (1.3-6.9)		NA	NA	NA	NA	NA	NA
No exposing agricultural occupations	24/206 (11.7)	1 [Reference]	.005	NA	NA	1 [Reference]	.001	NA	NA	NA	NA
Exposing agricultural occupations	11/38 (29.0)	3.1 (1.3-7.0)		NA	NA	5.1 (2.0-12.8)		NA	NA	NA	NA
No wood exposure	32/229 (14.0)	1 [Reference]	.46	NA	NA	NA	NA	NA	NA	NA	NA
Wood exposure	3/15 (20.0)	1.5 (0.4-5.8)		NA	NA	NA	NA	NA	NA	NA	NA
No green spaces exposure	32/228 (14.0)	1 [Reference]	.71	NA	NA	NA	NA	NA	NA	NA	NA
Green spaces exposure	3/16 (18.8)	1.4 (0.4-5.2)		NA	NA	NA	NA	NA	NA	NA	NA
No hygiene exposure	34/233 (14.6)	1 [Reference]	>.99	NA	NA	NA	NA	NA	NA	NA	NA
Hygiene exposure	1/11 (9.0)	0.6 (0.1-4.7)		NA	NA	NA	NA	NA	NA	NA	NA
No high probability of occupational exposure	23/196 (11.7)	1 [Reference]	.02	NA	NA	NA	NA	1 [Reference]	.005	NA	NA
High probability of occupational exposure	12/48 (25.0)	2.5 (1.1-5.5)		NA	NA	NA	NA	3.6 (1.5-8.5)	NA	NA	NA
No high probability and reliability of occupational exposure	27/209 (12.9)	1 [Reference]	.13	NA	NA	NA	NA	NA	NA	1 [Reference]	.02
High probability and reliability of occupational exposure	8/35 (22.9)	2.0 (0.8-4.9)		NA	NA	NA	NA	NA	NA	3.7 (1.4-10.1)	NA

Abbreviations: AOR, adjusted odds ratio; CNS, central nervous system; IPI, International Prognostic Index; NA, not applicable; OR, odds ratio.

^a^ For multivariate model, the sample size is 237 because of missing data for IPI in 7 patients.

^b^ Multivariate model 1 included variables: occupational exposure, IPI, and liver and lung determination. Hosmer-Lemeshow test, *P* > .99.

^c^ Multivariate model 2 included exposing agricultural occupations, IPI, lung determination, and bulky disease. Hosmer-Lemeshow test, *P* = .52.

^d^ Multivariate model 3 included high probability of occupational exposure, IPI, and lung determination. Hosmer-Lemeshow test, *P* = .74.

^e^ Multivariate model 4 included high probability and reliability of occupational exposure, IPI, liver and lung determination, and bulky disease. Hosmer-Lemeshow test, *P* = .84.

In multivariate analysis (Table 2), occupational exposure to pesticides remained independently associated with treatment failure (all occupational exposure vs no occupational exposure [model 1]: AOR, 3.0; 95% CI, 1.4-6.9; *P* = .009; exposing agricultural occupations vs others [model 2]: AOR, 5.1; 95% CI, 2.0-12.8; *P* = .001; high probability of occupational exposure vs others [model 3]: AOR, 3.6; 95% CI, 1.5-8.5; *P* = .005; high probability and reliability of occupational exposure vs others [model 4]: AOR, 3.7; 95% CI, 1.4-10.1; *P* = .02) (Table 2).

The probabilities and duration of exposure among exposed patients according to treatment response are given in eTable 2 in the Supplement.

### Secondary Outcomes

#### Event-Free Survival

**Figure 2.**
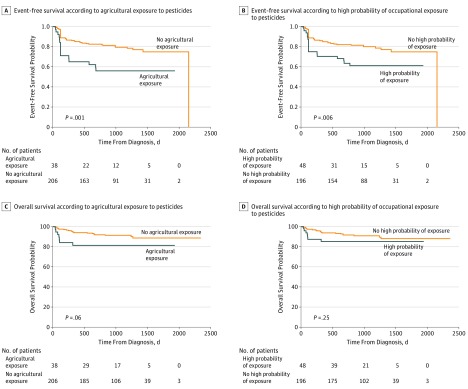
Event-Free and Overall Survival

In univariate analysis, unfavorable IPI, liver determination, lung determination, bulky disease, occupational exposure (Table 3), exposing agricultural occupation, and high probability and reliability of occupational exposure (Table 3 and Figure 2) were statistically associated with poorer EFS. Two-year event-free survival was 70% in the occupational exposed group vs 82% in the unexposed group (*P* = .04); among patients with exposing agricultural occupations compared with other patients, the difference was more pronounced (56% vs 83%; *P* = .002). In multivariate analysis, occupational exposure was associated with reduced EFS (all occupational exposure vs others: AHR, 2.2; 95% CI, 1.3-3.9; *P* = .005 [adjustment for IPI and liver determination]; agricultural occupations vs nonagricultural occupations: AHR, 3.5; 95% CI, 1.9-6.5; *P* < .001 [adjustment for IPI and lung determination]; high probability of occupational exposure vs others: AHR, 2.7; 95% CI, 1.5-4.9; *P* < .001 [adjustment for IPI and lung determination]; high probability and reliability of occupational exposure vs others: AHR, 2.6; 95% CI, 1.3-5.0; *P* = .006 [adjustment for IPI, liver, and lung determination]).

**Table 3.** Univariate Cox Proportional Hazards Regression Analysis for Overall Survival and Event-Free Survival With Known Risk Factors and Exposure to Pesticides

Characteristic	2-y Survival			2-y Event-Free Survival		
	Probability (95% CI)	Univariate HR (95% CI)	*P* Value	Probability (95% CI)	Univariate HR (95% CI)	*P* Value
Determination						
No lung	0.93 (0.88-0.95)	1 [Reference]	.002	0.81 (0.75-0.85)	1 [Reference]	.02
Lung	0.71 (0.48-0.85)	3.9 (1.6-9.3)		0.58 (0.36-0.75)	2.4 (1.2-4.8)	
No liver	0.92 (0.88-0.95)	1 [Reference]	.007	0.80 (0.74-0.85)	1 [Reference]	.006
Liver	0.74 (0.51-0.87)	3.5 (1.4-8.8)		0.61 (0.38-0.77)	2.6 (1.3-5.2)	
Bulky disease						
No	0.91 (0.86-0.94)	1 [Reference]	.70	0.82 (0.75-0.87)	1 [Reference]	.02
Yes	0.88 (0.76-0.94)	1.2 (0.5-2.8)		0.69 (0.55-0.79)	1.9 (1.1-3.3)	
IPI						
Favorable and intermediary I (0-2)	0.98 (0.93-0.99)	1 [Reference]	.001	0.90 (0.83-0.94)	1 [Reference]	<.001
Intermediary II (3)	0.89 (0.77-0.95)	4.8 (1.4-16.3)		0.68 (0.53-0.79)	4.6 (2.4-9.0)	
Unfavorable (4-5)	0.72 (0.56-0.83)	12.6 (4.1-38.7)		0.60 (0.44-0.73)	5.6 (2.8-11.3)	
Pesticide exposure						
No exposed occupation	0.92 (0.87-0.95)	1 [Reference]	.33	0.82 (0.75-0.87)	1 [Reference]	.04
All exposed occupation	0.86 (0.75-0.93)	1.5 (0.7-3.4)		0.70 (0.57-0.79)	1.8 (1.0-3.0)	
No exposing agricultural occupations	0.92 (0.87-0.95)	1 [Reference]	.07	0.83 (0.77-0.87)	1 [Reference]	.002
Exposing agricultural occupations	0.81 (0.65-0.91)	2.3 (0.9-5.3)		0.56 (0.38-0.70)	2.5 (1.4-4.4)	
No high probability of occupational exposure	0.92 (0.87-0.95)	1 [Reference]	.26	0.82 (0.76-0.87)	1 [Reference]	.009
High probability of occupational exposure	0.85 (0.72-0.93)	1.7 (0.7-4.0)		0.63 (0.48-0.76)	2.1 (1.2-3.7)	
No high probability and reliability of occupational exposure	0.91 (0.86-0.94)	1 [Reference]	.75	0.81 (0.75-0.86)	1 [Reference]	.08
High probability and reliability of occupational exposure	0.90 (0.85-0.93)	1.2 (0.4-3.5)		0.64 (0.45-0.78)	1.8 (0.9-3.6)	

Abbreviations: HR, hazard ratio; IPI, International Prognostic Index.

#### Overall Survival

 In univariate analysis, unfavorable IPI, liver determination, and lung determination were associated with poorer OS (Table 3); no pesticide exposure was associated with poorer OS (Table 3 and Figure 2). Overall survival was 81% in the agricultural exposed group vs 92% in the non–agricultural exposed group, *P* = .07. In multivariate analysis, occupational exposure was associated with reduced OS for exposed agricultural occupations vs all others (AHR, 3.9; 95% CI, 1.5-10.0; *P* < .001 [adjustment for IPI and lung determination]) but was not associated with reduced OS for high probability of occupational exposure vs all others (AHR, 2.4; 95% CI, 1.0-5.8; *P* = .06 [adjustment for IPI and lung determination]), all occupational exposure vs others (AHR, 1.8; 95% CI, 0.7-4.1; *P* = .19 [adjustment for IPI, liver determination]), or high probability and reliability of occupational exposure vs others (AHR, 2.1; 95% CI, 0.7-6.6; *P* = .19 [adjustment for IPI, liver, and lung determination]).

### Sensitivity Analysis

We performed a sensitivity analysis to examine the association of responder (patient or proxy), and the association was slightly increased in the subgroup of patient responders (n = 194, with 4 missing data for IPI) compared with the total population (eTable 3 in the Supplement). Multivariate analysis in the subgroup of proxy responders was not performed because of low size of sample (n = 44, with 3 missing data for IPI).

## Discussion

The results of this study suggest that the prognosis of DLBCL in terms of treatment failure and survival is statistically associated with a history of occupational exposure to pesticides, notably from agricultural occupations. Outcomes in our selected population and phase 3 study population are similar.^27,28^ Analysis of all preselected patients showed similar outcomes to those published in the real-life study for the use of R-CHOP to treat DLBCL.^12^ Contrary to the work of Le Guyader-Peyrou et al,^24^ we found no association between prognosis and living-alone status, travel time to hospital, and treatment in nonteaching hospitals, probably because of our study’s smaller sample size. There was a higher rate of an earlier stage at diagnosis among pesticide-exposed patients, which is traditionally associated with better prognosis (part of the IPI) and could bias the outcome; this variable was included in the multivariate model. The use of a JEM, which is more sensitive and less specific than job exposure modules based on specific tasks, is likely to attenuate the OR.^29^ We performed 4 analyses using JEM probability and reliability threshold for occupational exposure. The first analysis included all patients with low or high probability of exposure; another analysis considered only agricultural exposure, which is the better known according to the literature; another analysis considered high probability (>75%); and the last one considered high probability and reliability. There was an increasing gradient between the AOR for treatment failure for all probability of exposure (AOR, 3.0; 95% CI, 1.3-7.0), high probability (AOR, 3.6; 95% CI, 1.5-8.5) and high probability and reliability of exposure (AOR, 3.7; 95% CI, 1.4-10.1), and exposing agricultural occupations (AOR, 5.1; 95% CI, 2.0-12.8). This finding suggests that resistance to treatment could be provided by specific agents used in farming activities.

Standard treatment for DLBCL is 6 to 8 courses of R-CHOP, and interim response assessment after 4 courses has been found to be indicative of survival,^30^ leading to treatment change for patients not reaching complete response. Thus, response after 4 courses could be considered as a surrogate of chemosensitivity. No central assessment of response had been made in this retrospective study, and less than 5% of patients had computed tomographic evaluation, which could induce misclassification of incomplete responses. However, this bias seems to be limited because 2-year EFS produced similar results to treatment response.

### Limitations

Gene expression profiling has highlighted 2 groups of DLBCL: germinal center derived, with good prognosis, or activated B-cell derived.^11^ However, neither gene expression, germinal center, nor activated B-cell immunophenotyping was systematically performed in this study, and we could not evaluate the relationship between pesticide exposure and cell of origin.

Occupational history could only be assessed for 60.4% of the patients treated for DLBCL, mostly because of the high rate of deceased patients; thus, the reachable patients had a better prognosis, potentially overestimating or underestimating the adverse prognosis of occupational exposure to pesticides. Likewise, some interview responders are proxies, mostly because of deceased patients, with a worse outcome. We performed a sensitivity analysis on the patient responder subgroup, excluding proxy responders. The ORs were slightly increased among the patient responder subgroup. Selection and nondifferential misclassification bias are possible.

Retrospective occupational exposure assessment is difficult and leads to potential misclassification bias. Use of the PESTIPOP reduces nondifferential misclassification because of its high reproducibility.

PESTIPOP does not consider the nonprofessional intensive use of pesticides, sometimes spontaneously described by patients during the interview (eg, undeclared work on a family farm). Likewise, this study ignores exposure shorter than 1 year. However, JEMs are often the best way to estimate long-term exposures, especially in retrospective surveys when individuals are unable to remember or are unaware of past exposures.^31^ The association of professional exposure to pesticides with prognosis was stronger among patients with exposing agricultural occupations: the difference of response rate was higher than when considering all exposed patients, and the OR was higher. Pesticides exposure for other occupations based on PESTIPOP may be misclassifying unexposed as exposed because of the lack of pieces of information on specific tasks performed within the same jobs. In our study, no difference was found between short- and long-term exposure, but a patient sample of long-term exposed patients was small. We used logistic regression for response to immunochemotherapy, which estimates the relative risk with the OR, thus overestimating the relative risks. However, the estimated HRs for EFS analysis remained significant.

Another limitation was the consideration of pesticides as a cocktail of all different insecticides, fungicides, and herbicides used in farming, wood, hygiene, or green space activities as 1 homogeneous exposure for 40 years. Many different agents have been used, and another analysis of the results with PESTIMAT^32^ could give more precise details on the effects of single agents.

## Conclusions

This study suggests for the first time, to our knowledge, a poorer prognosis for patients with DLBCL exposed to pesticides, concerning the response to treatment, 2-year EFS, and OS. These findings must be confirmed in further prospective studies. The biology of these tumors and characterization of specific pesticides should also be studied.

## Notes

Supplement. **eAppendix.** Method for Matrix Generation **eTable 1.** Table of Comparison for Included and Not Included (Unreached or Refusal) Patients **eTable 2.** Table of Description of Pesticide Exposure Among Complete Response or Treatment Failure Exposed Patients **eTable 3.** Sensitivity Analysis: Subgroup of Patient Interview Responders
