# Supplementary material for: Association of Occupational Pesticide Exposure With Immunochemotherapy Response and Survival Among Patients With Diffuse Large B-Cell Lymphoma
Source: JAMA Netw Open. 2019 Apr 19;2(4):e192093. doi: 10.1001/jamanetworkopen.2019.2093 (PMC6481431; doi:10.1001/jamanetworkopen.2019.2093)
Supplement: Supplement. — eAppendix. Method for Matrix Generation eTable 1. Table of Comparison for Included and Not Included (Unreached or Refusal) Patients eTable 2. Table of Description of Pesticide Exposure Among Complete Response or Treatment Failure Exposed Patients eTable 3. Sensitivity Analysis: Subgroup of Patient Interview Responders [file jamanetwopen-2-e192093-s001.pdf]

Supplementary Online Content

Lamure S, Carles C, Aquereburu Q, et al. Association of occupational pesticide exposure with immunochemotherapy response and survival among patients with diffuse large B-cell lymphoma. *JAMA Netw Open*. 2019;2(4):e192093. doi:10.1001/jamanetworkopen.2019.2093

eAppendix. Method for Matrix Generation

eTable 1. Table of Comparison for Included and Not Included (Unreached or Refusal) Patients

eTable 2. Table of Description of Pesticide Exposure Among Complete Response or Treatment Failure Exposed Patients

eTable 3. Sensitivity Analysis: Subgroup of Patient Interview Responders

This supplementary material has been provided by the authors to give readers additional information about their work.

© 2019 Lamure S et al. *JAMA Network Open*

eAppendix. Method for Matrix Generation

A job-exposure matrix (JEM) is a table of correspondence between jobs and potential exposures. It automatically assigns an exposure to a subject extrapolated from professional history.

Matrices *a priori* are developed from the knowledge of multidisciplinary experts (epidemiologists, hygienists, occupational physicians, chemists). Matrices *a posteriori* are developed from a specific survey based on real situations based on the statistical exploitation of observations made in the field from a representative sample. "Mixed" matrices are developed from a survey completed and/or validated by a group of experts.

The PESTIPOP JEM aimed to assess pesticide occupational exposure in the general population. The matrix is composed of two axes: the first axis corresponding to occupations (combinations of occupations and industries) and the second one to pesticide exposure. The estimated exposure metric is the probability of exposure (proportion of exposed) coupled with a reliability assessment (low, medium or high). These metrics were defined by combining different sources, i) an *a priori* expert assessment (agricultural industry experts) and ii) data from the multicentre case control study on brain tumours in the general population CERENAT (occupational history, specific questionnaires); iii) a *a posteriori* expert assessment, based on the data of this case-control study.

eTable 1. Table of Comparison for Included and Not Included (Unreached or Refusal) Patients

Characteristics	Eligible, n=404	Included, n=244	Not included, n=160	*p*
Mean age, years +/-SD	64 +/-15	61+/-16	69 +/-14	0.07
Sex, n (%)				0.77
Men	251 (62.1)	153 (62.7)	98 (61.3)	
Women	153 (37.9)	91 (37.3)	62 (38.8)	
Previous cancer, n (%)				0.98
No	352 (87.1)	213 (87.3)	139 (86.9)	
Treated with RT/CT	18 (4.5)	11 (4.5)	7 (4.4)	
Not treated with RT/CT	34 (8.4)	20 (8.2)	14 (8.8)	
ECOG (n=393), n (%)				0.001
0	111 (27.5)	78 (32.5)	33 (21.6)	
1	148 (36.6)	99 (41.3)	49 (32.0)	
2	64 (15.8)	32 (13.3)	32 (20.9)	
3	38 (9.4)	18 (7.5)	20 (13.8)	
4	32 (7.9)	13 (5.4)	19 (12.4)	
Ann Arbor staging, (n=403), n (%)				0.001
I	71 (17.6)	56 (23.0)	15 (9.4)	
II	59 (14.6)	40 (16.4)	19 (12.0)	
III	53 (13.1)	31 (12.7)	22 (13.8)	
IV	220 (54.5)	117 (48.0)	103 (64.8)	
Determination, n (%)				
Bone marrow	41 (10.1)	22 (9.0)	19 (11.9)	0.35
Central nervous system	11 (2.7)	3 (1.2)	8 (5.0)	0.02
Lung	43 (10.6)	24 (9.8)	19 (11.9)	0.52
Liver	41 (10.2)	23 (9.4)	18 (11.2)	0.55
Gut	34 (8.4)	20 (8.2)	14 (8.6)	0.85

© 2019 Lamure S et al. *JAMA Network Open*

B symptoms, n (%)	103 (25.5)	59 (24.2)	44 (27.5)	0.45
Bulky disease, n (%)	92 (22.8)	59 (24.2)	33 (20.8)	0.42
LDH > N, n=234, n (%)	245 (60.6)	135 (55.3)	110 (68.8)	0.003
IPI, n=237, n (%)				<0.001
Favourable (0-1)	104 (25.7)	83 (35.0)	21 (14.1)	
Intermediary I (2)	87 (21.5)	55 (23.2)	32 (24.5)	
Intermediary II (3)	85 (21.0)	55 (23.2)	30 (20.1)	
Unfavourable (4-5)	110 (27.2)	44 (18.6)	66 (44.3)	
Transformation from low-grade lymphoma, n=387, n (%)	48 (11.9)	31 (13.2)	17 (11.1)	0.53
Main treatment				<0.001
R-CHOP	332 (82.2)	214 (87.7)	118 (73.8)	
R-miniCHOP	64 (15.8)	25 (10.3)	39 (24.4)	
R-ACVBP	7 (1.7)	5 (2.1)	2 (1.3)	
R-DA-EPOCH	1 (0.3)	0 (0)	1 (0.6)	
Teaching hospital				<0.001
yes<br>no	220 (54.5)<br>184 (45.5)	151 (68.6)<br>93 (38.1)	69 (43.1)<br>91 (56.9)	
Marital status				NA*
not alone	161 (81.3)	159 (82.0)	2 (50.0)	
alone	37 (18.7)	35 (18.0)	2 (50.0)	
unknown	206	50	156	
Travel time to hospital				0.003
< or =15 minutes	77 (19.2)	37 (15.2)	40 (25.5)	
16-44 minutes	219 (54.6)	138 (58.6)	81 (51.6)	
> or = 45 minutes	105 (26.2)	69 (28.3)	36 (22.9)	
unknown	3	0	3	
Evaluation				<0.001
Complete response	300 (74.3)	209 (85.2)	91 (56.9)	

© 2019 Lamure S et al. *JAMA Network Open*

PR/SD/Progression	79 (19.6)	31 (12.7)	48 (30)	
Toxic death	16 (4.0)	4 (1.6)	12 (7.5)	
Unknown	9 (2.2)	0 (0)	9 (5.6)	
2-year EFS (CI95%)	0.69 (0.64-0.73)	0.78 (0.72-0.83)	0.53 (0.45-0.61)	<0.001
2-year OS (CI95%)	0.79 (0.74-0.82)	0.90 (0.85-0.93)	0.60 (0.52-0.68)	<0.001

Abbreviations: SD=standard derivation, RT= radiotherapy, CT= chemotherapy, ECOG= European cooperative oncology group, LDH= lactate dehydrogenase, IPI= international prognostic index, EFS= event free survival, OS= overall survival, CI= confidence interval, PR= partial response, SD= stable disease

*: unreliable test because of missing data

© 2019 Lamure S et al. *JAMA Network Open*

eTable 2. Table of Description of Pesticide Exposure Among Complete Response or Treatment Failure Exposed Patients

**Probabilities and duration of exposure among exposed patients according to treatment response.**								
Characteristics	All exposed		Treatment failure			Treatment success		
	Mean (SD)	Median (IQR)	Mean (SD)	Median (IQR)	n	Mean (SD)	Median (IQR)	n
All exposed occupations (n=67)								
Probability of exposure	77.5 (37.8)	100 (50-100)	83.1 (35.6)	100 (100-100)	15	75.9 (38.6)	100 (50-100)	52
Duration of exposure	13.2 (13.6)	8 (2-22)	14.7 (18.3)	6 (2-28)	15	12.7 (12.1)	8.5 (3-21.5)	52
Exposing agricultural occupations (n=38)								
Probability of exposure (%)	99.3 (4.5)	100 (100-100)	100 (0)	100 (100-100)	11	99.0 (5.3)	100 (100-100)	27
Duration of exposure (years)	12.8 (14.8)	6 (2-17.5)	14.9 (20.9)	3 (2-37)	11	11.8 (11.6)	8 (3-16)	25
Wood exposure (n=15)								
Probability of exposure (%)	56.6 (43.5)	50 (7-100)	77.8 (38.5)	100 (33-100)	3	51.3 (44.6)	37.5 (7-100)	12
Duration of exposure (years)	12.6 (11.0)	10 (3-19)	10 (13.1)	4 (1-25)	3	13.3 (11.0)	11 (3-19)	11
Green space exposure (n=16)								
Probability of exposure (%)	43.1 (46.0)	7 (6-100)	37.5 (54.1)	7 (6-100)	3	44.4 (46.4)	7 (6-100)	13
Duration of exposure (years)	10.6 (11.7)	6 (2-15)	8.0 (7.2)	6 (2-16)	3	11.2 (12.7)	6 (2-14)	13

© 2019 Lamure S et al. *JAMA Network Open*

Hygiene exposure (n=11)								
Probability of exposure (%)	32.0 (32.3)	33 (4-50)	33.3 (-)	33 (-)	1	31.9 (34.1)	19 (4-50)	10
Duration of exposure (years)	7.0 (7.8)	2 (2-12)	2 (-)	2 (-)	1	7.5 (8.1)	2.5 (2-12)	10
Abbreviations: SD=standard derivation, IQR= interquartile range								

© 2019 Lamure S et al. *JAMA Network Open*

eTable 3. Sensitivity Analysis: Subgroup of Patient Interview Responders

Univariate and Multivariate logistic regression for treatment failure with occupational exposure, agricultural exposure, high probability of exposure and high probability and reliability of exposure, to pesticides in patients interview responders				
Characteristics	Univariate analysis, n=198		Multivariate analysis, n=194	
	OR (CI 95%)	p	OR_a (CI 95%)	p
Pesticide exposure				
No occupational exposure	1 (reference)		1 (reference)	
Occupational exposure	2.8 (1.0-7.9)	0.06	3.5 (1.2-10.3)*	0.03
No exposing-agricultural occupations	1 (reference)		1 (reference)	
Exposing agricultural eocupations	5.1 (1.7-15.0)	0.003	7.6 (2.2-25.8)**	0.002
No high probability of exposure	1 (reference)		1 (reference)	
High probability of exposure	3.5 (1.2-10.1)	0.02	4.2 (1.3-13.0)***	0.02
No high probability and reliability of exposure	1 (reference)		1 (reference)	
High probability and reliability of exposure	4.0 (1.3-11.9)	0.02	6.6 (1.9-23.3)****	0.004

Abbreviations: IPI= international prognostic index, OR = crude odds ratio, OR_a= adjusted odds ratio, CI= confidence interval
*Adjusting for IPI (0-2 vs 3 vs 4-5) and liver determination
** Adjusting for IPI (0-2 vs 3 vs 4-5), lung determination and Bulky disease
*** Adjusting for IPI (0-2 vs 3 vs 4-5) and lung determination
**** Adjusting for IPI (0-2 vs 3 vs 4-5),, liver and lung determination, and Bulky disease

© 2019 Lamure S et al. *JAMA Network Open*
